# Jolkinolide B induces apoptosis of colorectal carcinoma through ROS-ER stress-Ca^2+^-mitochondria dependent pathway

**DOI:** 10.18632/oncotarget.20077

**Published:** 2017-08-09

**Authors:** Jing Zhang, Yang Wang, Ye Zhou, Qing-Yu He

**Affiliations:** ^1^ Key Laboratory of Functional Protein Research of Guangdong Higher Education Institutes, Institute of Life and Health Engineering, College of Life Science and Technology, Jinan University, Guangzhou 510632, China

**Keywords:** jolkinolide B, iTRAQ proteomics, ROS, ER stress, Ca^2+^

## Abstract

Colorectal carcinoma (CRC) remains one of the leading causes of death in cancer-related diseases. In this study, we aimed to investigate the anticancer effect of Jolkinolide B (JB), a bioactive diterpenoid component isolated from the dried roots of *Euphorbia fischeriana* Steud, on CRC cells and its underlying mechanisms. We found that JB suppressed the cell viability and colony formation of CRC cells, HT29 and SW620. Annexin V/PI assay revealed that JB induced apoptosis in CRC cells, which was further confirmed by the increased expression of cleaved-caspase3 and cleaved-PARP. iTRAQ-based quantitative proteomics was performed to identify JB-regulated proteins in CRC cells. Gene Ontology (GO) analysis revealed that these JB-regulated proteins were mainly involved in ER stress response, which was evidenced by the expression of ER stress marker proteins, HSP90, Bip and PDI. Moreover, we found that JB provoked the generation of reactive oxygen species (ROS), and that inhibition of the ROS generation with N-acetyl L-cysteine could reverse the JB-induced apoptosis. Confocal microscopy and flow cytometry showed that JB treatment enhanced intracellular and mitochondrial Ca^2+^ level and JC-1 assay revealed a loss of mitochondrial membrane potential in CRC after JB treatment. The mitochondrial Ca^2+^ uptake and depolarization can be blocked by Ruthenium Red (RuRed), an inhibitor of mitochondrial Ca^2+^ uniporter. Taken together, we demonstrated that JB exerts its anticancer effect by ER stress-Ca^2+^-mitochondria signaling, suggesting the promising chemotherapeutic potential of JB for the treatment of CRC.

## INTRODUCTION

Colorectal carcinoma (CRC) is one of the most prevalent malignant cancers and the third leading cause of cancer-related deaths in the world [1]. Chemotherapy is the predominant therapeutic strategy used for the treatment of CRC patients, however, the 5-year survival rate of the patients was not substantially improved during the past 20 years [2]. Therefore, a critical need is to identify promising therapeutic molecules and to understand their action mechanisms against CRC for the further development of the potential drugs associated with a favorable clinical outcome.

The endoplasmic reticulum (ER) is a critical organelle for the protein folding, stress-sensing, Ca^2+^ storage and signaling transduction, and the main regulator in sustaining intracellular homeostasis [3]. Hypoxia, oxidative injury, or cytotoxic conditions can trigger ER stress responses by inducing the unfolded protein response (UPR) to maintain ER homeostasis; and when the stress signals are prolonged and unresolved, ER can initiate mitochondrial apoptosis [4]. As one of the most important second messengers in multiple cellular activities, calcium (Ca^2+^) released from the ER induces cell death mainly through the mitochondria-dependent apoptosis [5]. The influx of Ca^2+^ into mitochondria can disturb the Ca^2+^ homeostasis and the overloading of Ca^2+^ subsequently leads to the depolarization of mitochondria and apoptosis [6]. Therefore, the strategy of inducting UPR to target cancer cells has attracted great of interest, many potential drugs have shown promising outcome in inducing UPR and thus some of these drugs are under clinical trials [7].

Natural resource products have been used as sources of novel therapeutics for many years. To date, numerous natural plant ingredients possessing anticancer effects have received considerable attention in pharmacology studies [8, 9]. Jolkinolide B (JB) is a bioactive diterpenoid component isolated from the dried roots of *Euphorbia fischeriana* Steud. It has been reported that JB exhibited anti-adhesion and anti-invasion effects in human breast cancer MDA-MB-231 cells through the suppression of β1-integrin expression and the phosphorylation of focal adhesion kinase (FAK) [10]. Moreover, JB can induce apoptosis in human chronic myeloid leukemia [11, 12] *via* decreasing PI3K/Akt and the inhibitor of apoptosis protein (IAP) family proteins, and activating caspase-3 and -9. *In vitro* study has indicated that JB suppressed glycolysis by inhibiting the expression of glucose transporter genes and glycolysis-related kinase genes in melanoma [13], with the anti-tumor effect being solidly confirmed by mouse xenograft model. Due to its wide range of anti-tumor activities and low toxicity in animal models, JB probably is a promising chemotherapeutic agent for cancer therapy.

The rapid development of mass spectrometry technologies provides a powerful tool for accurate qualitative and quantitative proteomic analysis of cell signaling pathways [9, 14]. Sophisticated proteomic approaches have been widely used for the investigations of drug-action mechanism and drug target identification. In present study, we performed iTRAQ-based quantitative proteomics to study the anti-tumor effect of JB on colorectal cancer and found that JB could induce apoptosis in colorectal carcinoma *via* ROS-mediated ER stress and mitochondrial apoptotic pathways.

## RESULTS

### JB inhibits the growth of CRC cell lines

**Figure 1:**
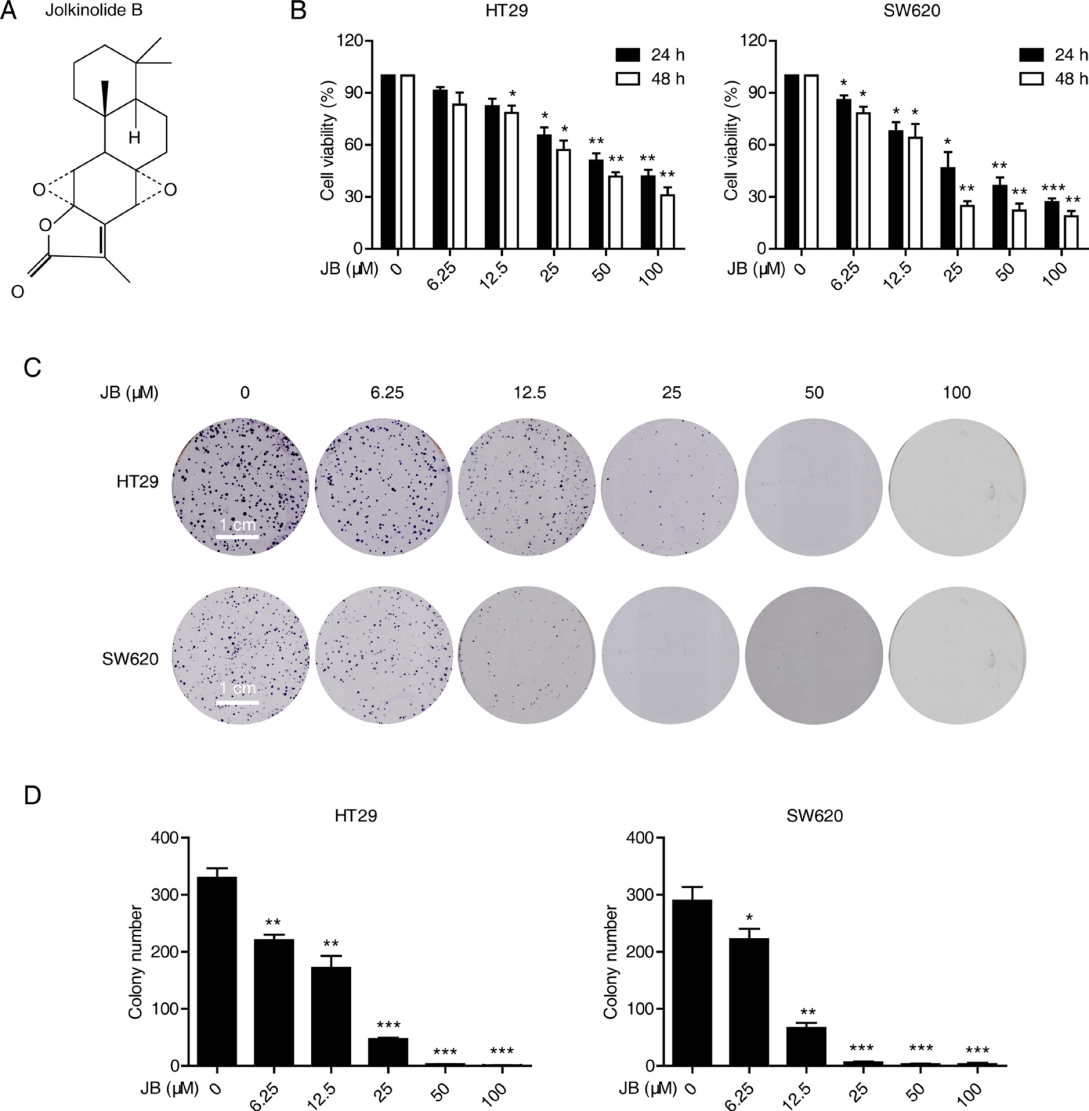
JB inhibits the growth of CRC cells (**A**) Chemical structure of JB. (**B**) HT29 and SW620 cells were incubated with indicated concentrations of JB for 24 and 48 h, the cell viability was determined by WST-1 assay. (**C**, **D**) HT29 and SW620 cells were treated with indicated concentrations of JB, Scale bar: 1 cm (C), and their abilities to form colonies were statistically presented in (D). Bars, SEM; *N* = 3; *P < 0.05, **P < 0.01, ***P < 0.001.

The chemical structure of Jolkinolide B is shown in Figure 1A. HT29 and SW620 are two representative CRC cell lines widely used for the investigation of anticancer agents [15, 16]. Here, we adopted these two cell lines for the following investigation. Firstly, HT29 and SW620 cells were treated with increasing concentrations of JB (0–100 µM) for 24 and 48 h, and the cell viability was determined by WST-1 assay. Figure 1B shows that JB inhibited the growth of HT29 and SW620 cells in dose- and time-dependent manners, with IC50 values of 59.78 ± 13.69 µM and 30.37 ± 7.61 µM after 24 h treatment, and 38 ± 3.34 µM and 18.25 ± 2.06 µM after 48 h treatment, respectively (Table 1). We also tested the cytotoxic effect of JB against normal cell lines including human colon epithelial cell line NCM460, human normal hepatocyte cell line LO2 and normal PBMC from two healthy volunteers by WST-1 assay. As shown in Table 1, JB induced little cytotoxic effect on these normal cell lines, with the IC50 values of more than 100 µM after 24 and 48 h treatment. Moreover, colony formation assay further confirmed the inhibitory effect of JB on the proliferation of both HT29 and SW620 cells. As shown in Figure 1C and 1D, colony formation ability of HT29 and SW620 cells was inhibited by JB in a dose-dependent manner. These data suggested that JB selectively inhibits the growth activity of CRC cells with minimal effects on normal cells, the following functional and mechanistic assays would therefore be performed with CRC cells only.

**Table 1:** Effects of Jolkinolide B on the viability of CRC cells and normal cells

Cell lines	IC50 (Mean ± SEM, µM)	
	24 h	48 h
HT29	59.78 ± 13.69	38.00 ± 3.34
SW620	30.37 ± 7.61	18.25 ± 2.06
NCM460	198.57 ± 18.81	127.37 ± 22.75
LO2	346.48 ± 55.48	297.33 ± 16.24
PBMC-1	286.86 ± 22.74	270.08 ± 19.71
PBMC-2	332.88 ± 15.05	132.78 ± 13.29

### JB induces apoptosis in CRC cells *via* caspase-dependent pathway

**Figure 2:**
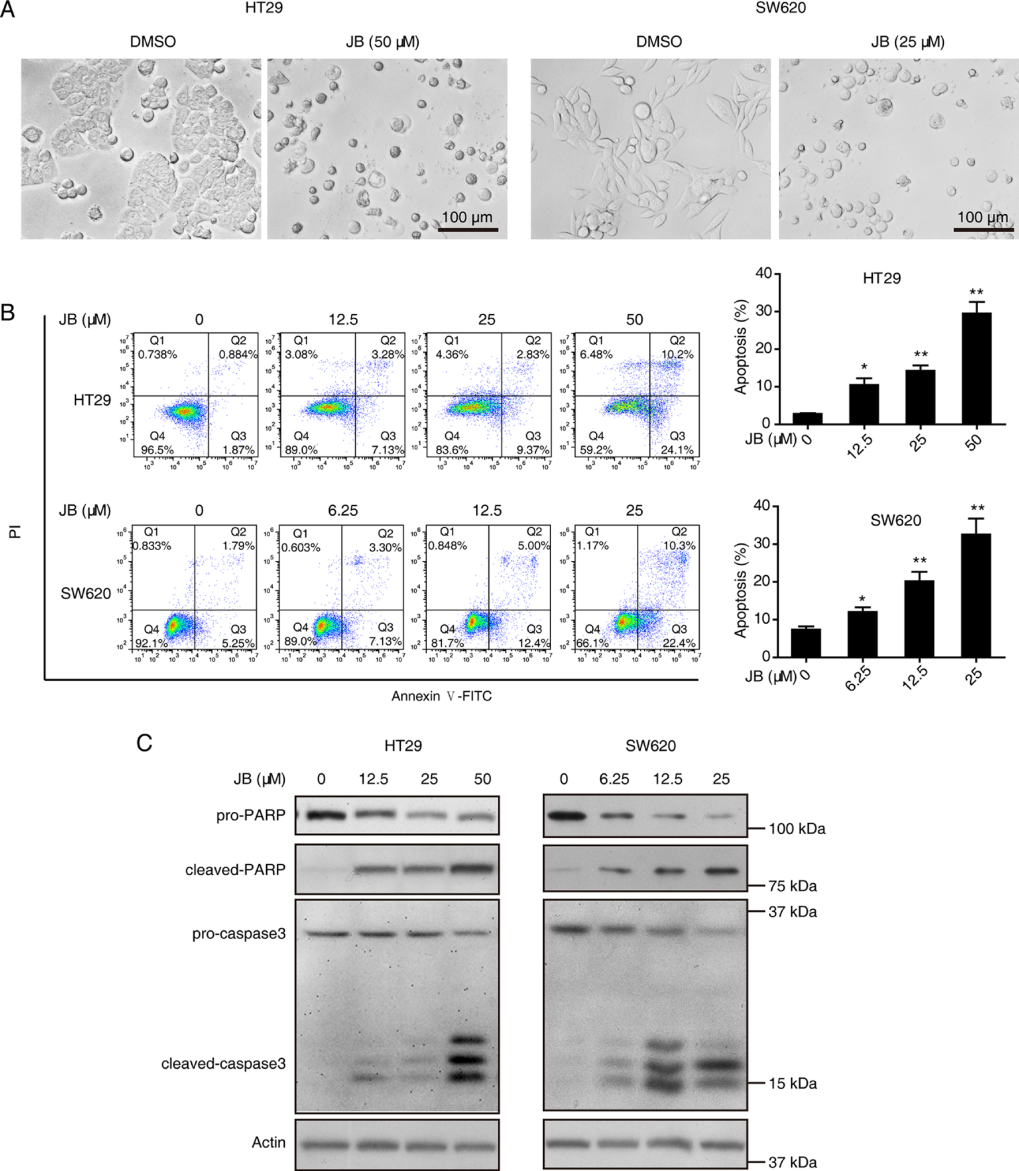
JB induces the apoptosis of CRC cells (**A**) HT29 and SW620 cells were treated with DMSO or JB for 24 h. Cell morphology was photographed under a microscopy (40 × magnification), Scale bar: 100 µm. (**B**) HT29 and SW620 cells were treated with different concentrations of JB for 24 h, the apoptotic cells were detected by Annexin V-FITC/PI double staining assay. The apoptotic cells including early apoptosis and late apoptosis were statistically presented, Bars, SEM; *N* = 3; *P < 0.05, **P < 0.01. (**C**) HT29 and SW620 cells were treated with various concentrations of JB for 24 h, the expression of apoptosis-related proteins including pro-PARP, cleaved-PARP, pro-caspase3, and cleaved-caspase3 were detected by immunoblotting.

Since JB exhibited significant inhibitory effect on the cell viability of HT29 and SW620, we asked whether JB induced apoptosis in CRC cells. Firstly, we observed the morphology of both HT29 and SW620 cells treated with JB for 24 h using a microscope. As shown in Figure 2A, significant cell shrinkage and decreased cellular attachment were observed in both HT29 and SW620 after JB treatment. Next, we incubated HT29 and SW620 cells with increasing concentrations of JB (up to 50 µM) for 24 h, the apoptotic cells were determined by Annexin V-FITC/PI double staining assay (Figure 2B), showing that apoptosis rate of HT29 and SW620 induced by JB increased in dose-dependent manners.

**Figure 3:**
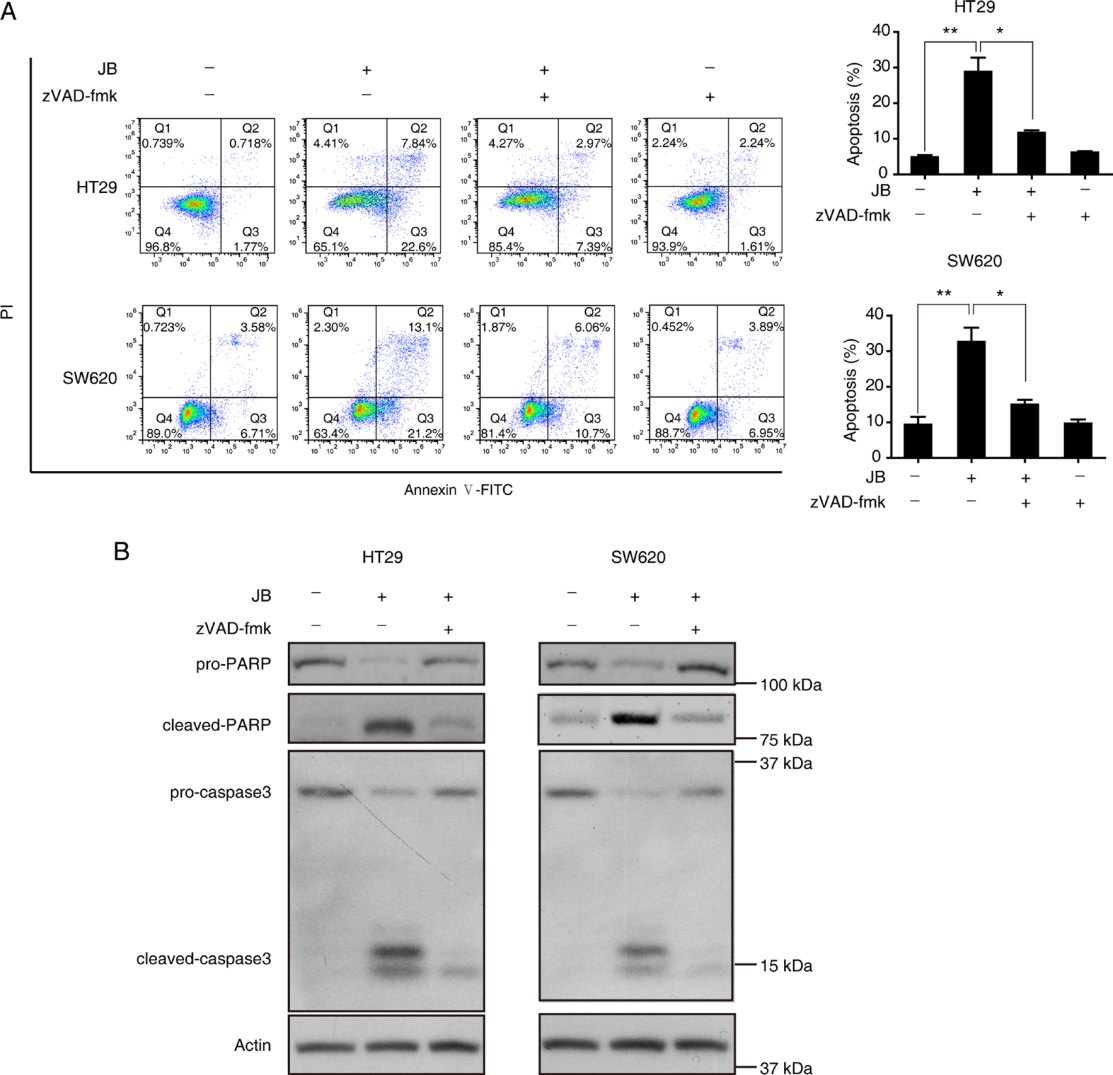
Caspase activation is required for the apoptosis induced by JB in CRC cells (**A**) HT29 and SW620 cells were treated with 50 µM or 25 µM JB for 24 h, respectively, in the absence and presence of 10 µM pan-caspase inhibitor z-VAD-fmk, then apoptotic cells were stained with Annexin V/PI and analyzed by flow cytometry. The apoptotic cells including early apoptosis and late apoptosis were statistically presented. Bars, SEM; *N* = 3; *P < 0.05, **P < 0.01. (**B**) HT29 and SW620 cells were treated with 50 µM or 25 µM JB for 24 h, respectively, in the absence and presence of 10 µM z-VAD-fmk, and the expression of apoptosis-related proteins including pro-PARP, cleaved-PARP, pro-caspase3, and cleaved-caspase3 were detected by immunoblotting.

To further delineate the mechanism by which JB induced apoptosis in HT29 and SW620 cells, Western blotting assay was performed, revealing that JB remarkably increased the expression of cleaved-caspase3 and cleaved-PARP in a dose-dependent manner (Figure 2C). Additionally, HT29 and SW620 cells were incubated with JB with or without the presence of z-VAD-fmk, a pan-caspase inhibitor, the results showed that z-VAD-fmk could effectively attenuate the JB-induced cell apoptosis (Figure 3A) and the expression of cleaved-caspase3 and cleaved-PARP (Figure 3B). These results demonstrated that JB could induce apoptosis in human colorectal cancer *via* the caspase-dependent pathway.

### iTRAQ-based proteomic analysis of CRC cells in response to JB treatment

**Figure 4:**
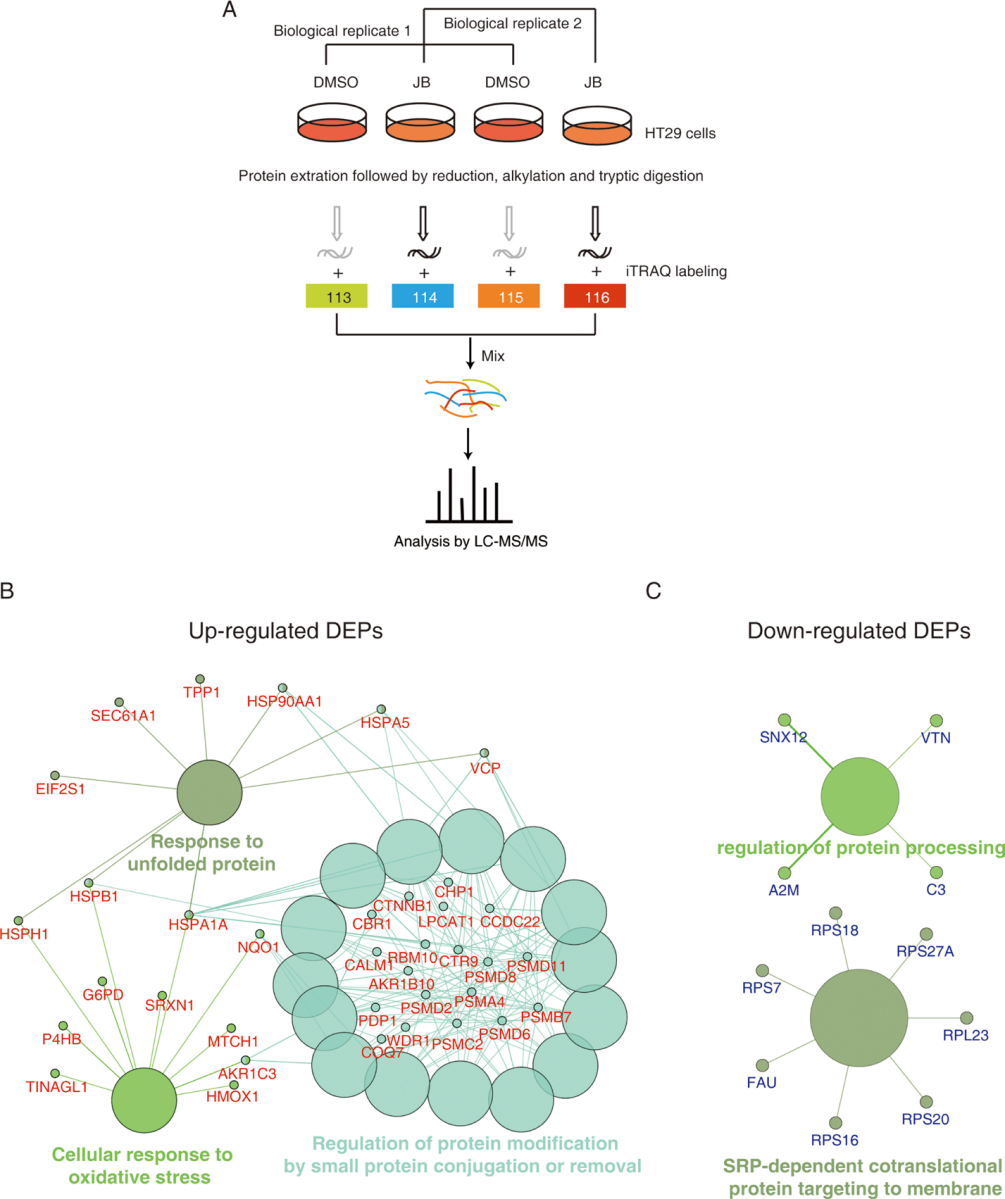
iTRAQ-based proteomics identifies JB-regulated proteins (**A**) Experimental flow chart of the identification of JB-regulated proteins. (**B**, **C**) DEPs were subjected to the Gene Ontology analysis by ClueGO+CluePedia software, up-regulated proteins (B) and down-regulated proteins (C) were enriched based on biological process, respectively.

To get insights into the mechanism of apoptosis induced by JB, we set up an iTRAQ-based quantitative proteomics to investigate the global protein change in JB-treated HT29 cells. The schematic diagram is shown in Figure 4A. HT29 cells treated with DMSO (Control) or JB (50 μM) for 24 h were subjected to LC-MS analysis *via* protein extraction, separation, tryptic digestion and iTRAQ labeling. A total of 3695 proteins with quantitative information were identified in the two replicated experiments (Supplementary Table 1). The proteins with a fold change > 1.5 or < 0.67 concurrently observed in the two experiments were considered to be significantly regulated. As such, 137 proteins were found to be up-regulated and 97 proteins were down-regulated (Supplementary Table 2).

To explore the biological progresses involved in the anticancer effects of JB in colon cancer cells, the significantly altered proteins were uploaded to ClueGO+CluePedia for GO analysis. As shown in Figure 4B, JB up-regulated proteins were significantly enriched in several biological progresses including response to unfolded protein, cellular response to oxidative stress, and regulation of protein modification by small protein conjugation or removal. When comparing the down-regulated proteins, we could enrich SRP-dependent co-translational protein targeting to membrane and regulation of protein processing (Figure 4C). The iTRAQ-based quantitative proteomic result suggested that ER stress as well as oxidative response may be the key event of JB-induced apoptosis in CRC.

### JB-induced ROS generation contributes to the ER stress and cell apoptosis

**Figure 5:**
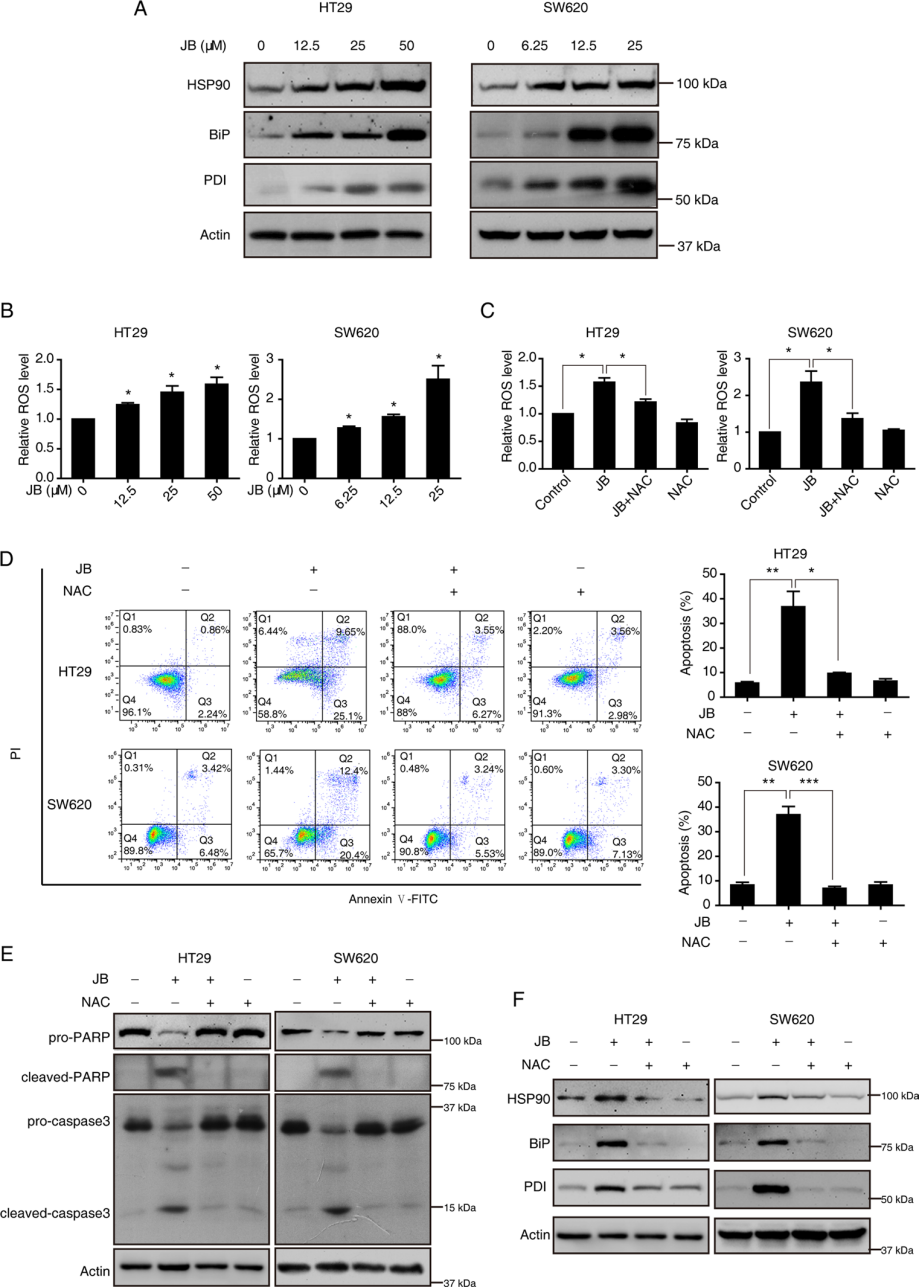
JB induces apoptosis *via* ROS/ER stress signaling (**A**, **B**) HT29 and SW620 cells were incubated with indicated concentrations of JB for 24 h, and the expression of ER stress-related proteins including HSP90, Bip and PDI were determined by Western blotting (A), the intracellular ROS was stained with dihydroethidium and measured by flow cytometry (B). (**C**–**F**) After pretreatment with or without 1 mM NAC for 2 h, HT29 and SW620 cells were treated with 50 or 25 µM JB for 24 h, and then the ROS (C), apoptosis (including early apoptosis and late apoptosis) (D), the expression of apoptotic markers (E) and ER stress-related markers (F) were analyzed; Bars, SEM; *N* = 3; *P < 0.05, **P < 0.01, ***P < 0.001.

Irreparable ER stress is a lethal signal for cancer cells suffering from high cytotoxicity [17]. According to our quantitative proteomic results, JB significantly induced ER stress and unfolded protein response in CRC cells, which probably contribute to the apoptosis induced by JB. We performed immunoblotting to verify several ER stress-associated markers including HSP90, Bip, and PDI, which were found up-regulated in the proteomic analysis. Our results showed that these proteins were remarkably increased in both HT29 and SW620 cells treated with JB for 24 h (Figure 5A).

It is well recognized that ROS is involved in ER stress and cell death [18], we then examined whether JB can induce ROS generation in CRC cells. Flow cytometric experiment using a ROS fluorescent probe Dihydroethidium demonstrated that the production of ROS was markedly increased after the treatment of JB for 24 h in both HT29 and SW620 (Figure 5B). As expected, pretreating the cells with NAC (1 mM), a ROS scavenger, significantly blocked the generation of ROS induced by JB (Figure 5C). Moreover, this pretreatment of NAC also attenuated JB-induced apoptosis (Figure 5D), as well as the expression of cleaved-PARP and cleaved-caspase3 (Figure 5E), suggesting a critical role of ROS in JB-induced apoptosis. Next, we also detected the effect of JB-induced ROS on the ER stress. As shown in Figure 5F, pretreatment of NAC could decrease the ER stress-associated markers, HSP90, BiP and PDI enhanced by JB, suggesting that JB-induced ROS generation acts as an upstream regulator of ER stress in the progress of cell apoptosis.

### JB-induced ER calcium release promotes the mitochondrial Ca^2+^ influx and mitochondria-dependent apoptosis

ER stress is accompanied by alteration in Ca^2+^ homeostasis, and the Ca^2+^ dysregulation can further promote cell death through apoptosis [5, 19]. Since mitochondria and ER are major reservoirs of intracellular Ca^2+^, we asked whether JB could induce apoptosis by perturbing intracellular Ca^2+^ homeostasis. At the beginning, we added indicated concentration of JB in HT29 and SW620 cells pre-labeled with Fluo-4AM (a cytosolic Ca^2+^ indicator), and monitored Fluo-4AM fluorescence (Supplementary Figure 1) within 600 s. As shown in Supplementary Figure 1A, JB-stimulation significantly evoked Ca^2+^ signal in the cytosol of HT29 and SW620 cells. Ionomycin and EGTA were respectively used as positive and negative controls (Supplementary Figure 1B and 1C).

**Figure 6:**
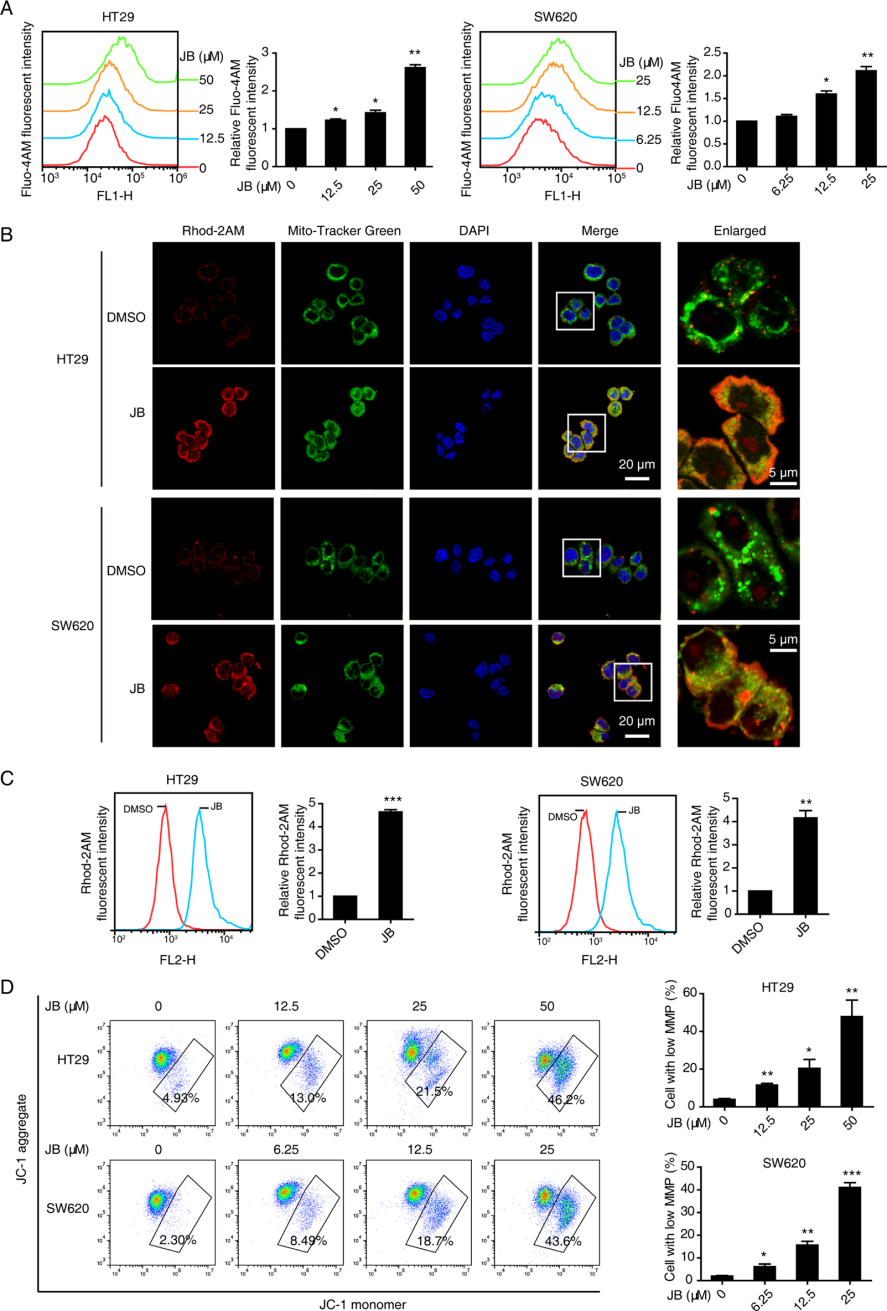
JB induces Ca^2+^ dysregulation and the loss of mitochondrial membrane potential (**A**) HT29 and SW620 cells were treated with indicated concentrations of JB for 24 h, the cytosolic Ca^2+^ was stained with Fluo-4AM and determined by flow cytometry. (**B**) HT29 and SW620 cells treated with DMSO or JB for 24 h were stained with 1 µM Rhod-2AM (red), a mitochondrial Ca^2+^ indicator dye, and 100 nM Mito-Tracker Green and then observed under confocal microscopy. Blue, DAPI. Scale bar = 20 μm; 5 μm (indicated enlargements). (**C**) The mitochondrial Ca^2+^ level alterations of HT29 and SW620 cells treated with DMSO or JB were determined by flow cytometry with Rhod-2AM staining. (**D**) The mitochondrial membrane potential levels of HT29 and SW620 treated with various concentrations of JB were evaluated by JC-1 assay, cells with low mitochondrial membrane potential were quantified. Bars, SEM; *N* = 3; *P < 0.05, **P < 0.01, ***P < 0.001.

Next, we detected cytosolic Ca^2+^ signal in CRC cells after 24 h of JB treatment. As shown in Figure 6A, incubation with JB dramatically enhanced the intracellular Ca^2+^ level in a dose-dependent manner. Meanwhile, we focused on the mitochondrial Ca^2+^ change by staining with Rhod-2AM, a mitochondrial Ca^2+^ indicator dye. Our confocal microscopy data showed that mitochondrial Ca^2+^ was increased in JB-treated cells as compared to untreated cells (Figure 6B). In addition, flow cytometry analysis supported the significance of increased mitochondrial Ca^2+^ level in CRC cells after JB treatment for 24 h (Figure 6C). Moreover, loss of mitochondrial membrane potential in both HT29 and SW620 cells was observed after 24 h of JB treatment (Figure 6D), where the cells with low mitochondrial membrane potential were quantified.

**Figure 7:**
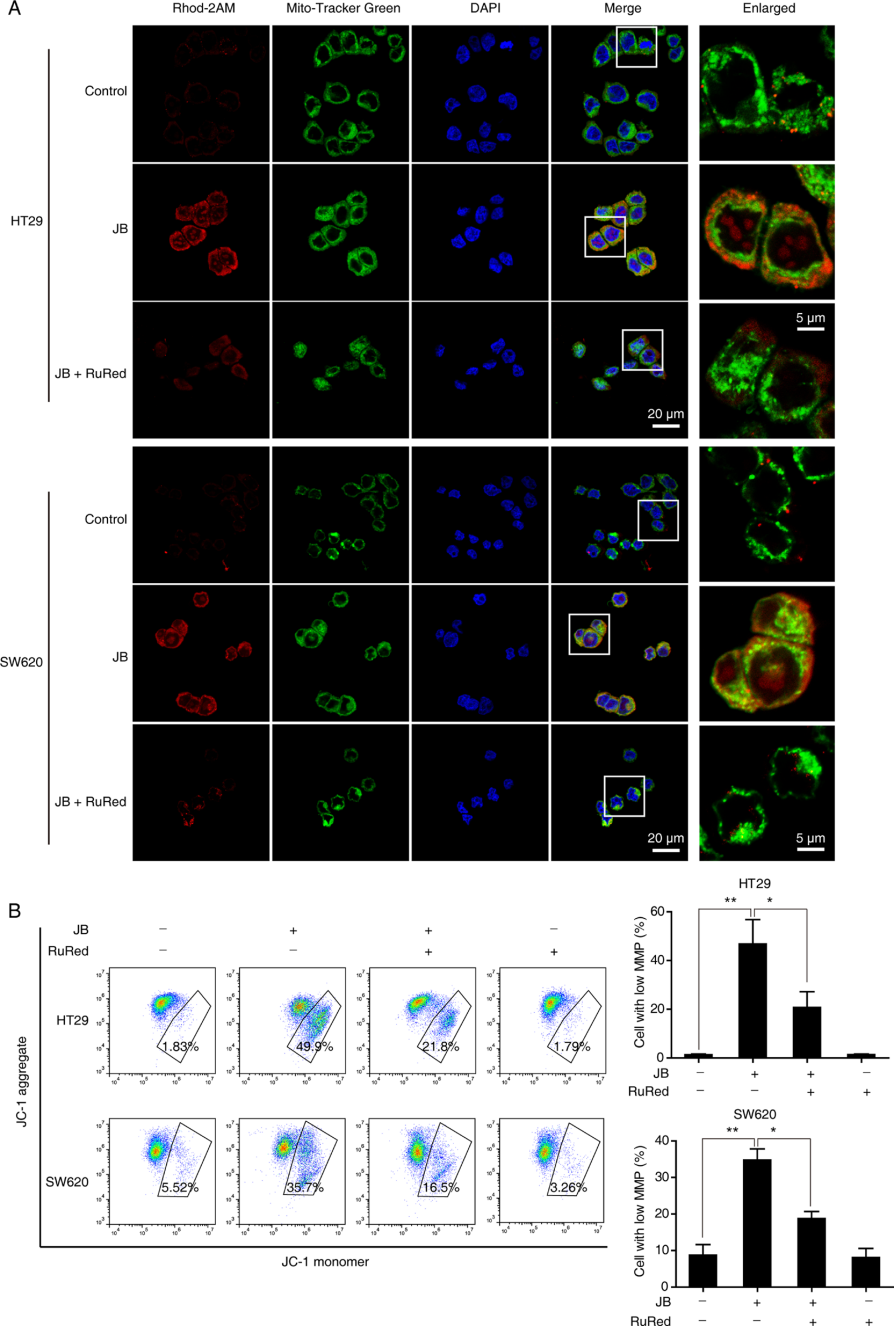
Mitochondrial Ca^2+^ influx is accountable for the loss of mitochondrial membrane potential (**A**) HT29 and SW620 cells pretreated with RuRed (2 μM) for 1 h were incubated with JB for 24 h, then the cells loaded with 1 µM Rhod-2AM (red), and 100 nM Mito-Tracker Green were observed under confocal microscopy. Blue, DAPI. Scale bar = 20 μm; 5 μm (indicated enlargements). (**B**) HT29 and SW620 cells were pretreated with 2 μM RuRed for 1 h and followed by JB treatment for 24 h, the mitochondrial membrane potential was determined by JC-1 assay. Cells with low mitochondrial membrane potential were quantified. Bars, SEM; *N* = 3; *P < 0.05, **P < 0.01.

To further confirm the critical role of mitochondrial Ca^2+^ influx in the loss of mitochondrial membrane potential, we tested whether the blockade of mitochondrial Ca^2+^ uptake could affect JB-induced mitochondrial depolarization. CRC cells pretreated with or without ruthenium red (RuRed), an inhibitor of uniporter-mediated mitochondrial Ca^2+^ uptake, were incubated with JB for 24 h, results showed that mitochondrial Ca^2+^ uptake was significantly attenuated after JB treatment (Figure 7A). As expected, RuRed pretreatment markedly inhibited the JB-induced loss of mitochondrial membrane potential (Figure 7B), suggesting that mitochondrial Ca^2+^ influx induced by JB may be the key molecular event in the mitochondrial depolarization, subsequently leading to apoptosis.

## DISCUSSION

CRC is a growing problem worldwide due to the rising incidences and lack of effective preventive agents. Thereby, the search for new anticancer agents that are more effective has attracted a great of interest. Phytochemicals from medicinal plants are important sources for cancer therapeutic drug discovery [20]. In past decades, many natural products including berberine [21], resveratrol [22] and quercetin [23] were found to exhibit significant anti-cancer effects and thus were applied in clinic. Jolkinolide B, a diterpenoid compound isolated from the dried plant roots of *Euphorbia Fscheriana* Steud, has previously been reported to have anticancer effects on cancer, such as inhibiting the proliferation of mouse melanoma [13], inducing the apoptosis of breast cancer [24, 25] and prostate cancer [26], and suppressing the metastatic activity of breast cancer cells [10]. As determined here, the IC50 of JB against CRC (18.25–38 µM, 48 h) is comparable to or smaller than those well-known promising agents including resveratrol (164.7 μM, 48 h) [27], quercetin (81.65 ± 0.49 μM, 48 h) [28] or some first line chemotherapy (5-FU, 92.85 µM, 72 h) [29], we speculate that JB could be a potential anticancer agent for CRC treatment.

Mechanistically, JB was found to inhibit PI3K/Akt/mTOR signaling pathway, leading to cell cycle arrest and apoptotic cell death [24]; however, the precise mechanisms underlying the apoptosis caused by JB in CRC have not been clearly determined. In the present work, we employed iTRAQ-based quantitative proteomics to profile the JB-regulated proteins in CRC cells and then evaluated the mechanistic clues indicated by the proteomic analysis. Our results showed that JB-induced cell apoptosis in colorectal carcinoma was associated with ER stress, which subsequently resulted in Ca^2+^ release and mitochondrial Ca^2+^ influx.

Mitochondria-mediated apoptosis is a classical intrinsic apoptotic pathway, deriving relevant drugs used for the elimination of malignant cancer in clinic. Given the high sensitivity of mitochondria/ER in cancer cells to oxidative stress and ER stress, as compared to normal cells, targeting ER-stress response may be a rational strategy for cancer therapy [30]. ER is a central cellular organelle that participates in a series of crucial biological progresses for sustaining cellular homeostasis. In the stimulation of irretrievable toxic stress, the ER can transmit lethal signals to execute cell death through mitochondrial pathway [17]. In this study, our iTRAQ proteomics identified a group of JB-regulated proteins relevant to “cellular response to oxidative stress”, which was reinforced by the stimulated expression of ER-stress markers (including HSP90, BiP and PDI) and ROS generation. These data demonstrated that JB can significantly induce ER stress in colon cancer.

Mitochondria and ER also are the major reservoirs of intracellular Ca^2+^, and the calcium signaling is one of the most critical aspects of the communication between the two organelles, in response to changing environmental conditions [31]. Mounting studies reported that Ca^2+^ release from ER is regulated by IP3R when cells suffer from prolonged ER stress [32], which elevates intracellular Ca^2+^ concentrations. The elevated cytosolic Ca^2+^ can evoke the activation of the Ca^2+^ uniporter and drive Ca^2+^ uptake to the mitochondria. Due to the relatively low affinity of the uniporter, long-lasting global cytosolic Ca^2+^ signals may result in the accumulation of vast amounts of Ca^2+^ in the mitochondria [33]. Large amounts of Ca^2+^ accumulated in the mitochondria will trigger apoptosis *via* Ca^2+^-mediated mitochondrial permeability transition [34]. Since ER stress was observed in our iTRAQ proteomic analysis, we further speculated that JB could induce apoptosis through Ca^2+^-mediated mitochondrial depolarization.

We used cytoplasmic Ca^2+^ specific cell permeable indicator Furo-4AM and mitochondrial Ca^2+^ indicator Rhod-2AM, respectively, to track Ca^2+^ flux in the cells treated with JB. As expected, JB treatment induced both the enhancement of cytosolic Ca^2+^ level and the mitochondrial colocalization of calcium signal, strongly indicating that mitochondrial Ca^2+^ uptake is a critical molecular event in JB-induced apoptosis.

Mitochondrial depolarization is a hallmark of the initiation process of the apoptotic cell death pathway. Intracellular Ca^2+^ overload or oxidative stress will eventually result in the breakdown of mitochondrial membrane potential, which causes caspase activation and downstream cascade effect [35, 36]. Our JC-1 assay supported that JB could induce mitochondrial depolarization in CRC cells, and the membrane potential could be restored in the pretreatment of RuRed (an inhibitor of mitochondrial Ca^2+^ uniporter). These data highlighted that JB-induced mitochondrial depolarization attributes to mitochondrial Ca^2+^ influx.

**Figure 8:**
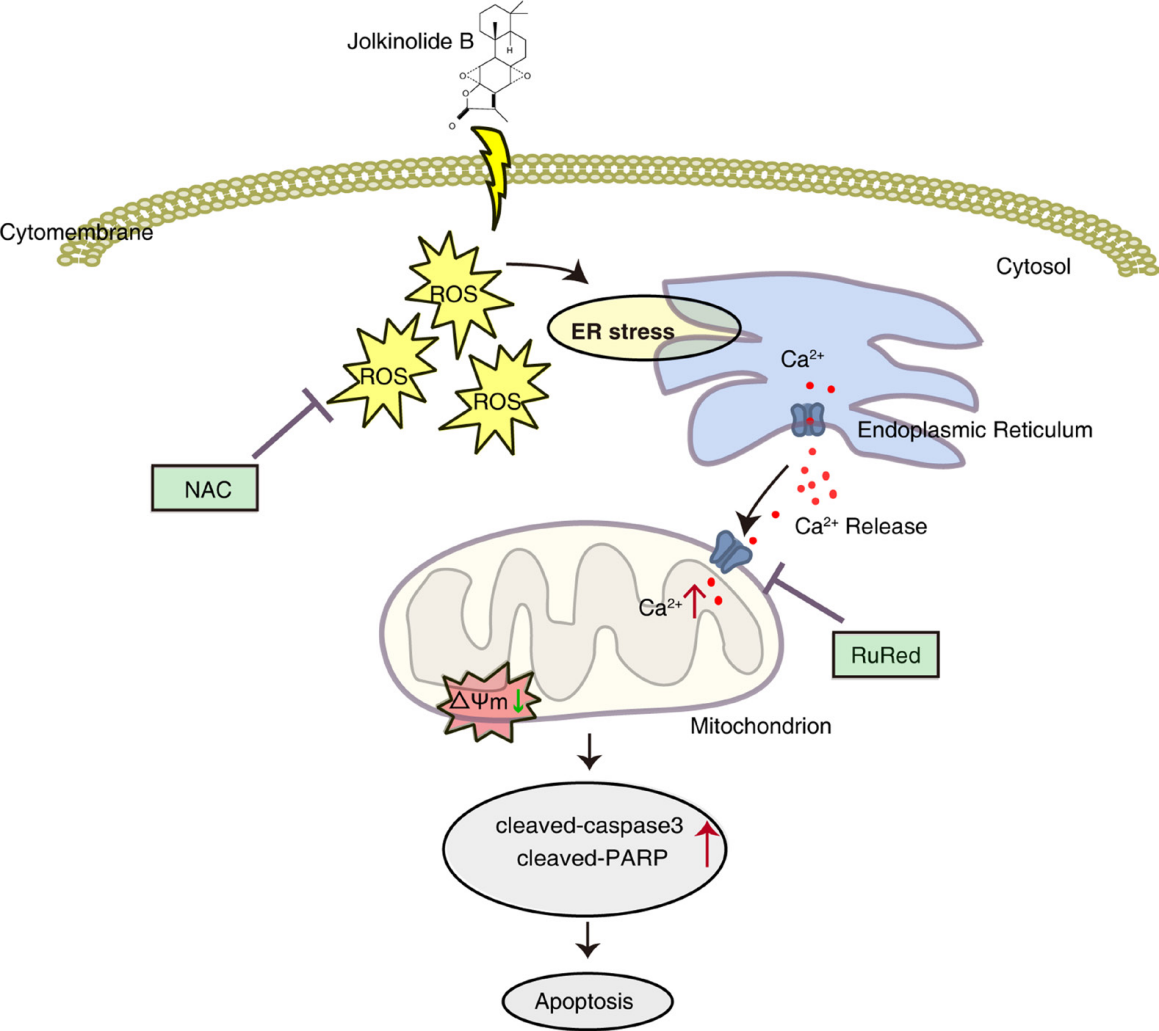
Schematic diagram of the apoptotic pathway induced by JB JB induces the generation of ROS, as well as ER stress, which promotes the release of Ca^2+^ from ER and subsequent mitochondrial Ca^2+^ uptake, leading to the dilations of mitochondrial depolarization and cell death in CRC cells.

In conclusion, we demonstrated that JB isolated from a medicinal plant possesses strong anticancer activity by inducing apoptosis in human colorectal carcinoma HT29 and SW620 cells. JB induces the generation of ROS and ER stress, which triggers Ca^2+^ release from the ER and mitochondrial Ca^2+^ uptake, leading to the dilations of mitochondrial depolarization and cell death (Figure 8). This finding provides solid evidence that JB may be a potential antitumor agent against colorectal carcinoma.

## MATERIALS AND METHODS

### Reagents and chemicals

Jolkinolide B (JB, ≥ 98% purity) was purchased from Must Bio-Technology Co. (Chengdu, China). Z-VAD-FMK was purchased from Selleckchem (Houston, TX, USA). *N*-acetyl-L-cysteine (NAC) and Dihydroethidium were purchased from Beyotime (Jiangsu, China). Ruthenium Red (RuRed) and ethylene glycol tetraacetic acid (EGTA) were purchased from Sigma (St.Louis, MO, USA). Ionomycin was from Merck Millipore (Billerica, MA, USA). Antibodies against pro-PARP, cleaved-PARP, pro-caspase3, cleaved-caspase3, BiP and PDI were obtained from Cell Signaling Technology (CST, Danvers, MA, USA). Antibodies against HSP90, β-actin and the HRP-conjugated goat anti-rabbit/mouse secondary antibody were purchased from Proteintech (Wuhan, Hubei, China).

### Cell lines and culture conditions

Human colorectal cancer cell lines HT29 and SW620 were obtained from the American Type Culture Collection (ATCC) and maintained in complete RPMI 1640 medium (Life Technologies, Beijing, China). Human peripheral blood mononuclear cells (PBMC) were isolated from the venous blood of two healthy adult volunteer donors by Ficoll-Hypaque gradient centrifugation and separated into individual lymphocyte cell types by fluorescence-activated cell sorting. Human colon epithelial cell line NCM460 (INCELL, San Antonio, TX, USA), human normal hepatocyte cell line LO2 (Type Culture Collection of the Chinese Academy of Sciences, Shanghai, China.) and PBMC were maintained in DMEM (Life Technologies). All culture media were supplemented with 10% fetal bovine serum (FBS, Gibco-Invitrogen Corporation, CA), 1% penicillin/streptomycin (GBCBIO Technologies, Guangzhou, China) and 10 μg/mL ciprofloxacin at 37°C.

### Cell viability assay

HT29 and SW620 cells were seeded in 96-well plates and cultured for 24 h. Then, cells were exposed to different concentrations of JB (ranged from 0 to 100 μM) for 24 h and 48 h. Cell viability was assessed by WST-1 assay as we described previously [14]. The concentration required to inhibit cell growth by 50% (IC50) was calculated by CalcuSyn (Version 2.11).

### Colony formation

Cells were seeded in 6-well plates at a density of 2000 cells per well and cultured for 48 h. Then, cells were exposed to different concentrations of JB (ranged from 0 to 100 μM) for 12 days to allow colony formation. The colonies were fixed with methyl alcohol for 15 min at room temperature and then stained with 1% crystal violet for 5 min. The number of colonies was counted with the ImageJ software (version 1.44I) and all statistical measurements were acquired from three independent experiments.

### Annexin V-FITC / PI staining assay

Apoptotic cells were determined by Annexin V-FITC/PI Apoptosis Detection Kit (Vazyme Biotech, Nanjing, China) according to the manufacturer’s protocol. In brief, cells were collected and suspended with 100 µL binding buffer and then stained with 5 µL FITC-conjugated Annexin V and 5 µL PI for 15 min at room temperature in dark place. Samples were analyzed *via* C6 flow cytometer (BD Biosciences, San Diego, CA). Data was analyzed quantitatively with FlowJo software (Version 7.6.5). Cell population in the Q4 quadrant (Annexin V-/PI- ) represents the live cells, population in the Q3 quadrant (Annexin V+/ PI- ) represents the early apoptotic cells, and population in the Q2 quadrant (Annexin V+/PI+) represents the late apoptotic or dead cells.

### Western blot analysis

Proteins were extracted from whole cells by RIPA lysis buffer (CST), followed by the concentration determination with a BCA kit (Thermo Fisher Scientific) as we previously described [14, 37]. The sample was then loaded onto a 10% or 12% SDS-PAGE and subsequently electrotransferred to a PVDF transfer membranes (Millipore, Bedford, MA, USA). The membrane was blocked for one hour with 5% nonfat milk. After blocking, the membrane was incubated with primary antibodies at 4 °C overnight, followed by appropriate secondary antibodies and then gel bands were visualized with the ECL reagent (BIO-RAD, Hercules, CA, USA).

### ROS detection

ROS was detected by staining the cells with Dihydroethidium as the manufacturers’ instructions. After incubation with the agent for 30 min, cells were collected and the fluorescent intensity was recorded by flow cytometric analysis.

### Ca^2+^ signals measurement

Cytosolic Ca^2+^ signals were measured with Fluo-4 AM (Beyotime) according to manufacturers’ instructions. In detail, cells were incubated with Fluo-4 AM (final concentration of 1 μM) for 30 min in PBS at 37 °C, then washed three times with PBS and incubated for an additional 15 min in the absence of Fluo-4AM to complete the de-esterification process of the dye. The fluorescent intensity was obtained by acquiring emission at 488 nm with flow cytometry.

The dynamic cytosolic Ca^2+^ was measured using the accuri C6 cytometer as reported previously [38]. In brief, HT29 and SW620 cells were labeled with Fluo-4 AM for 30 minutes at 37°C. The first 60 sec was recorded as baseline of calcium level, then, JB, ionomycin (1 μM) or EGTA (1 mM) were added and monitored up to 600 sec. Ionomycin was used as a positive control and EGTA was used as a negative control. Cytosolic calcium oscillations were presented as a ratio of F/F0 in the final results, where F is the fluorescence at any given time point and F0 represents baseline fluorescence intensity before treatment.

To measure the mitochondrial Ca^2+^ level, cells were incubated with 1 μM Rhod-2AM (Yeasen, Shanghai, China) for 20 min at 37 °C. The cells were then washed in PBS and the change in fluorescence was analyzed by flow cytometry. To further confirm the mitochondrial localization of Ca^2+^, cells were loaded with 1 μM Rhod-2AM for 20 min at 37 °C and then further loaded with 100 nM Mito-Tracker Green (Beyotime) for 20 min at 37°C. The cells were fixed with 4% paraformaldehyde before being mounted onto glass slides with Prolong Gold Anti-fade Reagent containing DAPI (Life Technologies) and observed with a Zeiss LSM710 confocal microscope as we described previously [9].

### Detection of mitochondrial membrane potential

The mitochondrial membrane potential was examined by staining the CRC cells with JC-1 (Beyotime), as the manufacturer’s instructions. Briefly, after the indicated treatments, cells were loaded with JC-1 for 20 min at 37°C. Then cells were collected and rinsed twice, followed by measuring fluorescent intensity on flow cytometer C6.

### iTRAQ-based quantitative proteomics

Proteins were extracted from the HT29 cells treated with DMSO or 50 μM JB for 24 h and subjected to isobaric tags for relative and absolute quantitation (iTRAQ) labeling using the AB SCIEX iTRAQ reagents multiplex kit according to the manufacturer’s instructions as we previously described [39, 40]. In detail, 200 μg proteins from each sample were first subjected to reduction and alkylation and then loaded in an ultracentrifuge filter (30-kDa; Sartorius Stedim Biotech, Shanghai, China), followed by two sequential buffer change centrifugations with 8 M urea and five-times volume of 50 mM NH_4_HCO_3_, respectively. Proteins were digested overnight at 37°C with trypsin, and peptides were centrifuged into the bottom tube for collection. Then a pair of DMSO and JB treated peptide samples were labeled with iTRAQ labeling reagent 113 and 114, respectively, and likewise another pair of biological replicates of the same samples (whole cell lysate from another batch of culture of these two treatments) were labeled with 115 and 116 iTRAQ labeling reagents, respectively. After incubation at room temperature for 2 h, the labeled peptides were combined and dried using vacuum centrifugation. The combined iTRAQ-labeled peptides were fractionated by high-pH RP-LC (10 fractions) and then analyzed with a triple TOF 5600 MS (5600 MS; AB SCIEX, Framingham, CA, USA) as we previously described [41].

### Database search and bioinformatics analyses

The wiff. MS data files were searched against Uniprot-Swiss Human database (2016_01 Release, 20193 entries) using ProteinPilot software 4.5 (AB SCIEX) with Paragon algorithm. User defined search parameters were set as we previously described [39, 40] : (1) Sample Type, iTRAQ 4plex (Peptide Labeled); (2) Cys. Alkylation, Iodoacetic acid; (3) Digestion, Trypsin; (4) Instrument, Triple-TOF 5600; (5) ID Focus, Biological modifications; (6) Search effort, Thorough; (7) Detected Protein Threshold [Unused ProtScore (Conf)] > 1.30 (95.0%).

Differentially expressed proteins (DEPs) were analyzed against the GO Biological Process in the Cytoscape (v3.4.0) software and performed in ClueGO v2.3.2 + CluePedia v1.3.2 plug-in as we described previously [42, 43].

### Statistical analysis

All grouped data were presented as mean ± S.E.M. from three independent experiments and statistical significance was determined using a two-tailed Student’s *t*-test. All statistical analyses were performed using GraphPad Prism software (version 5.01, San Diego California USA, www.graphpad.com). *P* < 0.05 was considered statistically significant.

## SUPPLEMENTARY MATERIALS FIGURE AND TABLES






