# Supplementary material for: Jolkinolide B induces apoptosis of colorectal carcinoma through ROS-ER stress-Ca2+-mitochondria dependent pathway
Source: Oncotarget. 2017 Aug 9;8(53):91223–37. doi: 10.18632/oncotarget.20077 (PMC5710918; doi:10.18632/oncotarget.20077)
Supplement: Supplementary file 1 [file oncotarget-08-91223-s001.pdf]

Jolkinolide B induces apoptosis of colorectal carcinoma through ROS-ER stress-Ca^{2+}-mitochondria dependent pathway

SUPPLEMENTARY MATERIALS

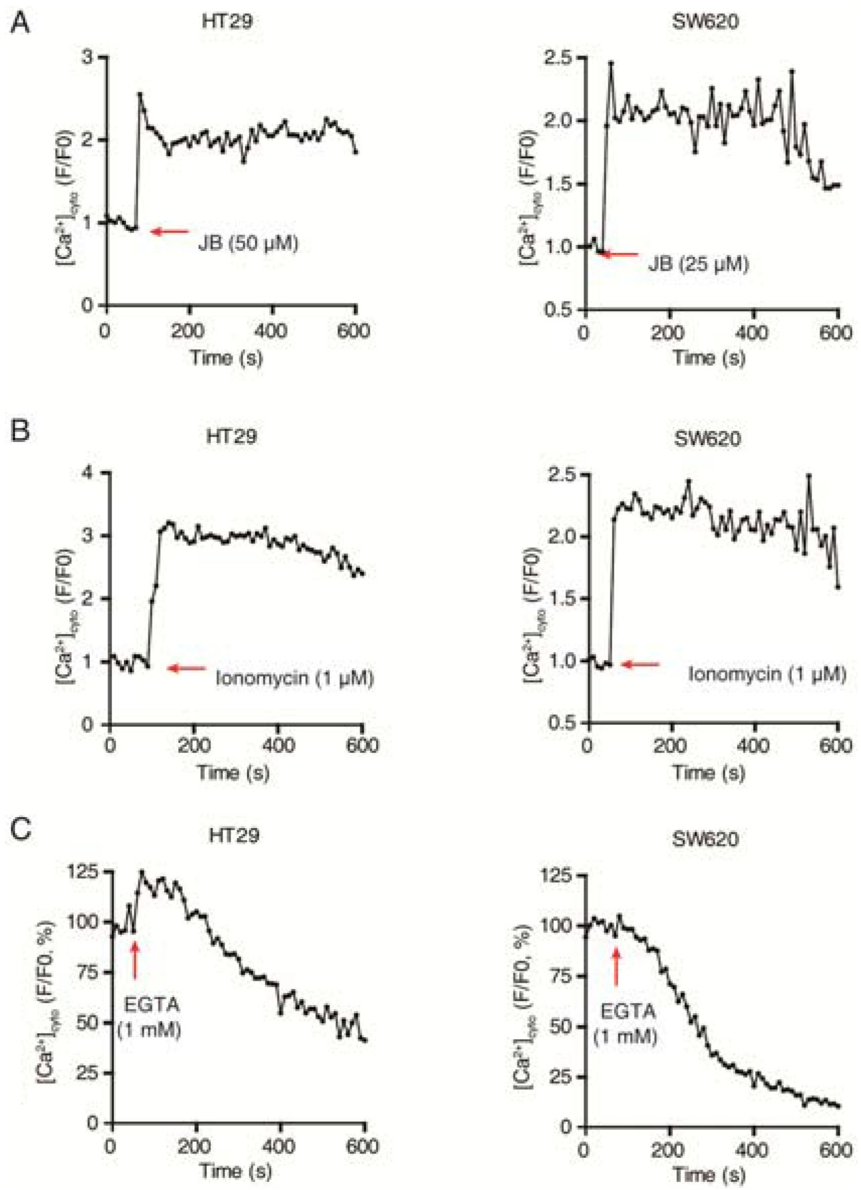

Supplementary Figure 1: Dot plot of fluorescence data of Fluo-4AM versus time displaying the changes of cytosolic Ca^{2+} signaling in CRC cells. (**A–C**) HT29 and SW620 cells were loaded with Fluo-4AM dyes. A base line reading was taken by flow cytometer for 60 sec followed by treatment with JB (50 µM for HT29 and 25 µM for SW620), ionomycin (1 µM) or EGTA (1 mM). Changes in cytosolic Ca^{2+} were recorded per 10 sec and presented as F/F0 ratio using Rhod-4AM fluorescence data.

Supplementary Table 1: Overview of protein identifications and quantifications for two biological experiments. See Supplementary_Table_1

Supplementary Table 2: Differentially expressed proteins in HT29 treatment with JB vs DMSO comparison. See Supplementary_Table_2
